# Safety and preliminary efficacy of Aurora: a pilot, non-randomized clinical trial of a culturally adapted digital cognitive behavioral therapy intervention for anxiety and depression in Mexico

**DOI:** 10.3389/fpsyt.2026.1824079

**Published:** 2026-04-28

**Authors:** Edith Zárate, Augusto César Velasco-Téllez, Lucia Araceli Sanchez-Reyes, Ángel Mario Coll-Muñoz, Miguel Ángel Ramírez-Ramírez, Alejandro López-Tello, Christian Gabriel Toledo-Lozano, Ana Moreno-Coutiño, María del Pilar Callejas-Gómez, Raúl Durón-Figueroa, Lorena Alejandra Flores-Plata, Antonio Ramirez-Treviño, Sebastian Nava-López, Diego Antonio Ocampo-Gutiérrez de Velasco, Oscar Arias-Carrión

**Affiliations:** 1 Psicofarma S.A. de C.V., Mexico City, Mexico; 2 Fundación FelizMente, Mexico City, Mexico; 3 Servicios Especializados en Ensayos Clínicos, Mexico City, Mexico; 4 Departamento de Psicología, Universidad Iberoamericana, Mexico City, Mexico; 5 Coordinación de Investigación, Centro Médico Nacional “20 de Noviembre”, ISSSTE, Mexico City, Mexico; 6 Laboratorio de Atención Plena Compasiva, Facultad de Psicología, Universidad Nacional de México, Mexico City, Mexico; 7 Private Practitioner, Mexico City, Mexico; 8 Laboratorio de Enseñanza Virtual y Ciberpsicología, Facultad de Psicología, Universidad Nacional Autónoma de México, Mexico City, Mexico; 9 Facultad de Estudios Superiores Iztacala, Universidad Nacional de México, Mexico City, Mexico; 10 CINVESTAV Unidad Guadalajara, Zapopan, Jalisco, Mexico; 11 División de Neurociencias Clínica, Instituto Nacional de Rehabilitación Luis Guillermo Ibarra Ibarra, Mexico City, Mexico; 12 Tecnologico de Monterrey, Escuela de Medicina y Ciencias de la Salud, Mexico City, Mexico

**Keywords:** anxiety disorders, digital cognitive behavioral therapy, digital therapeutics, generalized anxiety disorder, mobile health intervention

## Abstract

**Background/objective:**

Anxiety and depressive disorders are leading causes of disability worldwide, and access to evidence-based psychological treatment remains limited in many middle-income countries. Digital cognitive–behavioral therapy (CBT) interventions have emerged as scalable tools to address this treatment gap, yet few have undergone clinical evaluation in Latin American populations. This study aimed to assess the safety and preliminary efficacy of Aurora, a Spanish-language, culturally adapted digital CBT program, when used as an adjunct to pharmacotherapy in adults with generalized anxiety disorder.

**Methods:**

In a multicenter, open-label, non-randomized pilot study, 34 adults diagnosed with generalized anxiety disorder receiving stable pharmacological treatment were assigned through pragmatic, convenience-based allocation either to an experimental group (Aurora plus medication; n = 24) or to a control group receiving medication alone (n = 10). The sample had a mean age of 39.85 ± 12.88 years, with a predominance of women (22/34). Participants were followed for 12 weeks with assessments at baseline and weeks 4, 8, and 12. Clinical outcomes included anxiety severity measured by the Generalized Anxiety Disorder-7 (GAD-7), pathological worry assessed by the Penn State Worry Questionnaire (PSWQ), and depressive symptoms evaluated using the Patient Health Questionnaire-9 (PHQ-9). Safety was monitored through structured adverse-event reporting. Statistical analyses included linear mixed-effects models for longitudinal outcomes, ordinal logistic regression for severity transitions, and negative binomial regression and Fisher’s exact test for adverse events, with false discovery rate correction applied where appropriate.

**Results:**

Aurora demonstrated a favorable safety profile, with no serious adverse events and comparable adverse-event incidence between groups under structured clinical monitoring at weeks 4, 8, and 12. Anxiety symptoms (GAD-7) showed a significant effect of time (F_3,96_ = 169.65; p < 0.001), indicating reductions across both groups. Pathological worry (PSWQ) demonstrated significant group (F_1,31.12_ = 6.96; p = 0.013) and group × time interaction effects (F_3,93.4_ = 7.86; p < 0.001), with greater reductions in the Aurora group, particularly at weeks 8 and 12. At week 12, ordinal analyses indicated higher odds of lower worry severity in the intervention group (β = 2.53; p = 0.004; OR = 12.5). Depressive symptoms decreased similarly in both groups. Positive effect increased progressively across intervention modules, and module-embedded cognitive measures of anxiety and depression showed significant reductions over time.

**Conclusion:**

This pilot study provides preliminary, hypothesis-generating evidence that a culturally adapted digital CBT intervention can be safely integrated with pharmacotherapy and may be associated with enhanced improvements in anxiety-related outcomes, particularly pathological worry, in a Mexican clinical population. However, the non-randomized design, small sample size, and baseline imbalances limit causal inference and generalizability, and findings should be interpreted with caution. Larger randomized controlled trials are needed to confirm efficacy, determine long-term clinical impact, and guide the implementation of digital therapeutics in Latin American mental health systems.

## 1 Introduction

Anxiety and depressive disorders remain among the leading contributors to global disability, accounting for more than 10% of all years lived with disability and affecting over 600 million individuals worldwide (1). Despite decades of evidence supporting cognitive behavioral therapy (CBT) and modern antidepressants, a persistent treatment gap continues to undermine progress: more than 70% of people with common mental disorders receive no adequate care in low- and middle-income countries (LMICs), and even in upper-middle-income countries (UMICs), access remains highly unequal (2). Structural limitations—including scarce specialist workforce, fragmented services, stigma, and financial barriers—exert disproportionate effects on vulnerable populations, necessitating new models of scalable, evidence-based care.

Mexico exemplifies these challenges. With lifetime prevalence estimates of 14.3% for anxiety and 9.2% for depression (3), national demand for mental health care vastly exceeds specialist availability, with fewer than three psychiatrists per 100,000 inhabitants (4). The COVID-19 pandemic amplified these unmet needs, intensifying psychological distress while disrupting routine access to in-person services (5). Although classified as a UMIC, Mexico’s mental health infrastructure mirrors constraints faced across Latin America, where high out-of-pocket costs and limited psychotherapy availability hinder continuity of care (6). This convergence of high prevalence, limited access, and rising demand underscores an urgent need for scalable, culturally adapted interventions.

Digital therapeutics—technology-enabled interventions delivering structured, evidence-based treatment—represent a promising strategy to expand access in resource-constrained settings. Smartphone-based CBT programs have demonstrated consistent efficacy in reducing anxiety and depressive symptoms across diverse populations (7, 8). Recent meta-analyses further show that digital CBT can achieve effect sizes comparable to face-to-face treatment, particularly when engagement is high (9, 10). Importantly, emerging evidence from LMICs indicates that culturally adapted digital interventions can be feasible, acceptable, and clinically impactful, even among individuals with limited access to traditional psychotherapy (11–13). Nevertheless, despite a proliferation of mental health apps, the vast majority lack rigorous clinical validation, regulatory oversight, or grounding in established therapeutic models (14, 15). This gap signals both an opportunity and an imperative: to combine robust scientific methods with human-centered design to produce digital therapeutics capable of addressing real-world clinical needs.

Aurora was developed to meet this challenge. It is a Spanish-language, self-guided mobile application that delivers structured CBT through interactive modules focused on core evidence-based components, including cognitive restructuring, behavioral activation, mindfulness-based strategies, and emotional regulation techniques. Its development followed a co-design, human-centered approach, integrating clinicians, patients, and digital health experts to ensure therapeutic fidelity, usability, and cultural relevance. A preceding feasibility study in Mexico demonstrated high usability, favorable engagement, and moderate pre–post reductions in anxiety and depression (16). These findings highlighted the app’s potential to address persistent treatment gaps in Spanish-speaking populations.

Therefore, a critical next step is to determine whether a digital CBT intervention such as Aurora can meaningfully augment pharmacotherapy in routine psychiatric care. Evidence suggests that blended or hybrid approaches—combining digital interventions with medication—may accelerate symptom improvement, enhance self-management skills, and support long-term maintenance (17, 18). Translationally, digital therapeutics may target mechanisms not fully addressed by pharmacological treatment, including cognitive biases, perseverative worry, avoidance behavior, and deficits in positive affect—domains that have been shown to predict both treatment response and long-term prognosis (19–21).

In this context, we conducted a pilot, non-randomized, controlled clinical study to evaluate the safety and preliminary efficacy of Aurora as an adjunct to stable pharmacotherapy among adults with generalized anxiety disorder and clinically relevant depressive symptoms. Building on the feasibility and usability evidence from the initial Aurora study, our objectives were threefold: 1) to assess the safety and tolerability of combining a digital CBT intervention with standard pharmacological treatment; 2) to examine longitudinal trajectories of anxiety, pathological worry, and depressive symptoms, including severity-level transitions of clinical relevance; and 3) to explore psychological process indicators such as positive affect and cognitive symptom fluctuations, which may illuminate pathways through which digital interventions confer benefit.

We hypothesized that (i) Aurora would demonstrate a favorable safety and tolerability profile when combined with pharmacotherapy; (ii) participants receiving Aurora would show greater reductions over time in anxiety (GAD-7) and pathological worry (PSWQ) compared with pharmacotherapy alone; and (iii) the intervention would be associated with improvements in depressive symptoms (PHQ-9) and positive affect trajectories, reflecting underlying cognitive–emotional changes targeted by CBT.

Taken together, this study aims to provide an initial clinical evaluation of a culturally adapted digital therapeutic within routine psychiatric care, contributing to the development of scalable, data-informed, and patient-centered interventions for mental health systems in Latin America and other resource-constrained settings.

## 2 Methods

### 2.1 Study design

This study was a multicenter, open-label, non-randomized pilot clinical trial designed to evaluate the safety and preliminary efficacy of Aurora. The trial was conducted across two private outpatient psychiatry clinics in Mexico. Each participant completed a 12-week treatment period, with assessments at baseline and weeks 4, 8, and 12. Because the primary aim was to characterize safety and feasibility, no formal hypothesis testing or sample-size calculation was performed. The sample size was determined pragmatically to ensure minimal viable operational capacity and to provide sufficient information for the design of future randomized trials. Participants were allocated to either an experimental group receiving Aurora in addition to standard pharmacotherapy or a control group receiving pharmacotherapy alone, through pragmatic, convenience-based allocation, reflecting real-world clinical implementation.

### 2.2 Participants

Adults aged 18 years or older were eligible if a board-certified psychiatrist diagnosed generalized anxiety disorder (GAD) according to DSM-5-TR criteria and if their baseline GAD-7 score was ≥10. Participants were required to provide written informed consent and to use a smartphone-based digital therapeutic.

Key exclusion criteria included recent exposure to psychiatric medication within the prior six months, comorbid substance use disorders, acute psychiatric emergencies, bipolar disorder, seizure history, significant cardiovascular, hepatic, or renal disease, and pregnancy or breastfeeding. Individuals already engaged in CBT at study entry or unable to comply with study demands were also excluded.

Psychiatric diagnoses and comorbidities were established by board-certified psychiatrists using DSM-5-TR criteria, supported by structured clinical interviews and medical history reviews. Substance use and physical health conditions were assessed through clinical evaluation and baseline medical assessment. Participants were recruited from two outpatient psychiatry clinics, where treating psychiatrists identified eligible individuals and referred them to the study team during routine consultations.

### 2.3 Interventions

#### 2.3.1 Aurora digital CBT program

Aurora is a twelve-week, self-guided digital CBT intervention delivered via smartphone (16). The program comprises a basal module followed by eight sequential modules incorporating psychoeducation, cognitive restructuring, behavioral strategies, guided exercises, and multimedia resources.

The intervention is grounded in core evidence-based CBT components, including cognitive restructuring, behavioral activation, mindfulness-based strategies, and emotional regulation techniques, and was developed using a co-design, human-centered framework involving clinicians, patients, and digital health experts (16).

Each module includes five Likert-type items assessing positive affect and, in modules 0 and 8, brief anxiety- and depression-related cognitive items used as exploratory psychological process indicators. The experimental group received training in Aurora use at baseline and retained access to the program through week 12.

#### 2.3.2 Pharmacotherapy

All participants, regardless of group, received standard pharmacotherapy beginning at baseline. Escitalopram (10 mg/day) was initiated as first-line treatment during the first four weeks. Based on clinical response and tolerability, treatment was sequentially adjusted, including dose escalation to 20 mg/day or switching to duloxetine (30–60 mg/day). This approach reflects guideline-recommended, real-world management of generalized anxiety disorder and comorbid depressive symptoms. Concomitant medications and adherence were documented at each visit.

### 2.4 Study procedures and assessments

Screening procedures included medical history, physical examination, and baseline anxiety measurement using the GAD-7 scale. At the baseline visit (week 0), participants completed the GAD-7, Penn State Worry Questionnaire (PSWQ), and Patient Health Questionnaire-9 (PHQ-9), and were assigned to the intervention or control group.

Follow-up visits were scheduled for weeks 4, 8, and 12. At each visit, clinicians recorded vital signs, adverse events, treatment adherence, and any modifications to pharmacotherapy. Participants again completed the GAD-7, PSWQ, and PHQ-9. Use of the Aurora modules was automatically logged within the app. At week 12, participants underwent a final clinical evaluation, adverse-event review, and device return.

Safety was systematically assessed at each visit through structured clinical interviews, including active elicitation and classification of adverse events according to severity, expectedness, and relationship to the intervention. Predefined criteria for serious adverse events and psychiatric emergencies were applied to guide discontinuation and ensure patient safety.

### 2.5 Outcomes

#### 2.5.1 Primary safety outcomes

Safety was defined by the incidence, severity, and relatedness of adverse events recorded throughout the 12-week study. Serious adverse events and psychiatric emergencies were predefined as criteria for discontinuation.

#### 2.5.2 Clinical outcomes

Symptom change in anxiety, pathological worry, and depression was assessed using the GAD-7, PSWQ, and PHQ-9 at weeks 0, 4, 8, and 12. Severity categories for each scale were also evaluated at week 12 to examine clinically meaningful shifts in symptom profiles.

#### 2.5.3 Exploratory psychological process outcomes

Aurora’s in-app assessments provided additional exploratory variables. Positive emotions ratings from all nine modules (0–8) were used to characterize affective trajectories during treatment.

These variables were analyzed as exploratory, process-level outcomes distinct from the intervention description and reflecting underlying cognitive–emotional mechanisms targeted by digital CBT.

Brief cognitive items capturing anxiety- and depression-related thoughts were collected in modules 0 and 8 to explore whether changes reflected broader cognitive shifts associated with clinical improvement.

### 2.6 Ethical considerations

The study adhered to the principles of the Declaration of Helsinki and to national ethical standards governing clinical research in Mexico. Ethical approval for all procedures—including recruitment, assessment, data handling, and delivery of the digital intervention—was granted by the Comité de Ética en Investigación (Folio CEI-000002) and the Comité de Investigación (Folio CI-000002) of Médica Sur, S.A.B. de C.V., under protocol INNOVA 2024-01. All participants provided written informed consent prior to enrollment.

As the intervention involved a non-invasive, self-guided digital therapeutic without pharmacological manipulation, the trial posed minimal physical risk; nonetheless, continuous safety monitoring was implemented throughout.

### 2.7 Data analysis

#### 2.7.1 Safety monitoring and adverse-event analysis

Safety was evaluated at each study visit using structured elicitation of adverse events (AEs). All AEs were documented and classified by severity, expectedness, and attribution to the intervention, consistent with emerging regulatory guidance for behavioral and Software-as-a-Medical-Device (SaMD) therapeutics.

Because AE counts were sparse and overdispersed, we applied negative binomial regression to compare AE incidence between the experimental and control groups. Results were expressed as incidence rate ratios (IRRs). To complement this rate-based analysis, the proportion of participants experiencing ≥1 AE was compared using Fisher’s exact test.

#### 2.7.2 Longitudinal analysis of anxiety, pathological worry, and depressive symptoms

Trajectories of anxiety (GAD-7), pathological worry (PSWQ), and depressive symptoms (PHQ-9) were examined over 12 weeks using linear mixed-effects models (LMMs). Repeated measures were obtained at baseline and at weeks 4, 8, and 12. Each model included fixed effects for group, time, and their interaction, as well as a random intercept to account for within-participant correlation.

This modelling framework inherently accounts for baseline variability and within-subject trajectories over time, reducing bias associated with initial group differences.

Type III ANOVA was used to evaluate fixed effects. To compare groups at each assessment, estimated marginal means were derived, followed by false-discovery-rate (FDR) adjusted contrasts.

#### 2.7.3 Analysis of clinical severity levels

Because categorical shifts in clinical severity often carry greater decision-making relevance than changes in mean scores alone, we conducted complementary ordinal analyses. For each scale, the week-12 severity category was modeled using cumulative-link (ordinal logistic) regression, yielding odds ratios reflecting the likelihood of assignment to a lower (more favorable) severity category in the experimental group. Fisher’s exact test was performed on group-by-severity contingency tables to provide a distributional, nonparametric confirmation of group differences.

These analyses were designed to capture clinically meaningful changes beyond statistical significance.

#### 2.7.4 Positive emotional states across intervention modules

To investigate changes in positive affect—a proposed mechanistic pathway in digital CBT (22)—we analyzed repeated ratings of five positive-emotion items across nine modules (0–8). LMMs were fitted with fixed effects for module, question, and their interaction, along with subject-level random intercepts. When the interaction was not significant, a reduced model with only main effects was selected via a likelihood-ratio test.

To explore preliminary psychological process associations, we generated participant-level slopes representing change over time for positive emotions and for each clinical scale (GAD-7, PSWQ, PHQ-9). Pearson correlations were then computed to evaluate whether improvements in positive affect co-occurred with reductions in anxiety, worry, or depressive symptoms.

#### 2.7.5 Module-level assessments of anxiety- and depression-related cognitions

Two additional LMMs were used to assess changes in anxiety- and depression-related items embedded within the modules. Each model included module as a fixed effect and both item and subject as random effects to allow for item heterogeneity and repeated measurement. Type III ANOVA evaluated temporal change.

Concurrent validity was examined by correlating module-derived anxiety and depression scores with corresponding clinical scales at baseline, week 12, and for pre–post change scores.

No imputation procedures were applied, as analyses were conducted on complete-case data with minimal missingness, consistent with the exploratory nature of the pilot study.

## 3 Results

A total of 34 participants were enrolled: 24 were assigned to the experimental group and 10 to the control group, following pragmatic, convenience-based allocation consistent with the exploratory, non-randomized design of this pilot study (**Table 1**). All participants completed the 12-week follow-up, with minimal missing data; analyses were therefore conducted on complete-case data without imputation.

**Table 1:** Sociodemographic and baseline clinical characteristics of participants in the Aurora pilot trial.

Characteristic	Total sample (N = 34)	Experimental (n = 24)	Control (n = 10)	P-value
Age, years	39.85 ± 12.88 [18–59]	39.17 ± 13.14 [18–59]	41.50 ± 12.77 [18–59]	0.638
Sex, n (%)	22 F (64.7%)/12 M (35.3%)	12 F (50.0%)/12 M (50.0%)	10 F (100%)/0 M (0%)	0.006**
BMI, kg/m²	25.57 ± 4.54 [18–37]	25.69 ± 4.74 [18–37]	25.28 ± 4.25 [18–32]	0.817
Baseline anxiety (GAD-7)	15.50 ± 3.25 [9–21]	14.92 ± 3.11 [11–21]	16.90 ± 3.32 [9–20]	0.106
Baseline worry (PSWQ)	65.85 ± 7.98 [49–80]	65.61 ± 6.03 [50–80]	66.40 ± 11.70 [49–80]	0.798
Baseline depressive symptoms (PHQ-9)	16.76 ± 6.00 [6–27]	16.83 ± 5.61 [6–27]	16.60 ± 7.18 [7–27]	0.920
GAD-7 severity, n (%)				0.005**
Minimal	0 (0%)	0 (0%)	0 (0%)	
Mild	1 (2.9%)	0 (0%)	1 (10.0%)	
Moderate	11 (32.4%)	11 (45.8%)	0 (0%)	
Severe	22 (64.7%)	13 (54.2%)	9 (90.0%)	
PSWQ severity, n (%)				0.149
Low	0 (0%)	0 (0%)	0 (0%)	
Moderate	5 (14.7%)	2 (8.3%)	3 (30.0%)	
High	28 (82.4%)	21 (87.5%)	7 (70.0%)	
PHQ-9 severity, n (%)				0.406
Minimal	0 (0%)	0 (0%)	0 (0%)	
Mild	3 (8.8%)	1 (4.2%)	2 (20.0%)	
Moderate	12 (35.3%)	10 (41.7%)	2 (20.0%)	
Moderately severe	7 (20.6%)	5 (20.8%)	2 (20.0%)	
Severe	12 (35.3%)	8 (33.3%)	4 (40.0%)	

Values are presented as mean ± standard deviation with ranges in brackets [min–max]. Categorical variables are presented as counts and percentages.

Between-group comparisons were performed using independent-samples t-tests for continuous variables and Fisher’s exact test for categorical variables.

**p < 0.01 (APA-style significance notation).

The significant difference in sex distribution between groups (p = 0.006) reflects baseline imbalance inherent to the non-randomized, convenience-based allocation and should be considered when interpreting outcomes.

Severity categories were defined according to standard cut-offs: GAD-7 (minimal: 0–4; mild: 5–9; moderate: 10–14; severe: ≥15), PSWQ (low, moderate, high based on validated thresholds), and PHQ-9 (minimal: 0–4; mild: 5–9; moderate: 10–14; moderately severe: 15–19; severe: ≥20).

### 3.1 Safety

The intervention demonstrated a favorable safety profile under systematic, structured clinical monitoring at weeks 4, 8, and 12. No deaths or serious adverse events were observed. Six participants (17.6%) reported a total of nine mild, self-limited adverse events that were considered expected—nausea (n=4), flatulence (n=3), and constipation (n=2). All events occurred during mid-treatment visits and resolved without medical intervention or discontinuation of treatment.

Between-group comparisons revealed no evidence of differential AE burden. Negative binomial regression showed comparable AE incidence in the experimental and control groups (IRR 0.69; p=0.71), and the proportion of participants experiencing at least one AE did not differ significantly (Fisher’s exact p=1.00; OR = 1.24, 95% CI 0.09–10.87). These findings should be interpreted cautiously, given the small sample size, but support a preliminary favorable safety profile of the digital intervention when used alongside standard pharmacotherapy.

### 3.2 Anxiety trajectories (GAD-7)

Across the 12-week intervention, anxiety symptoms declined markedly in both groups (Week effect: F_3,96_=169.65; p<0.001). Although neither the main Group effect (p=0.061) nor the Group × Week interaction (p=0.085) reached statistical significance, both suggested a modest trend toward greater improvement in the experimental arm.

**Figure 1:**
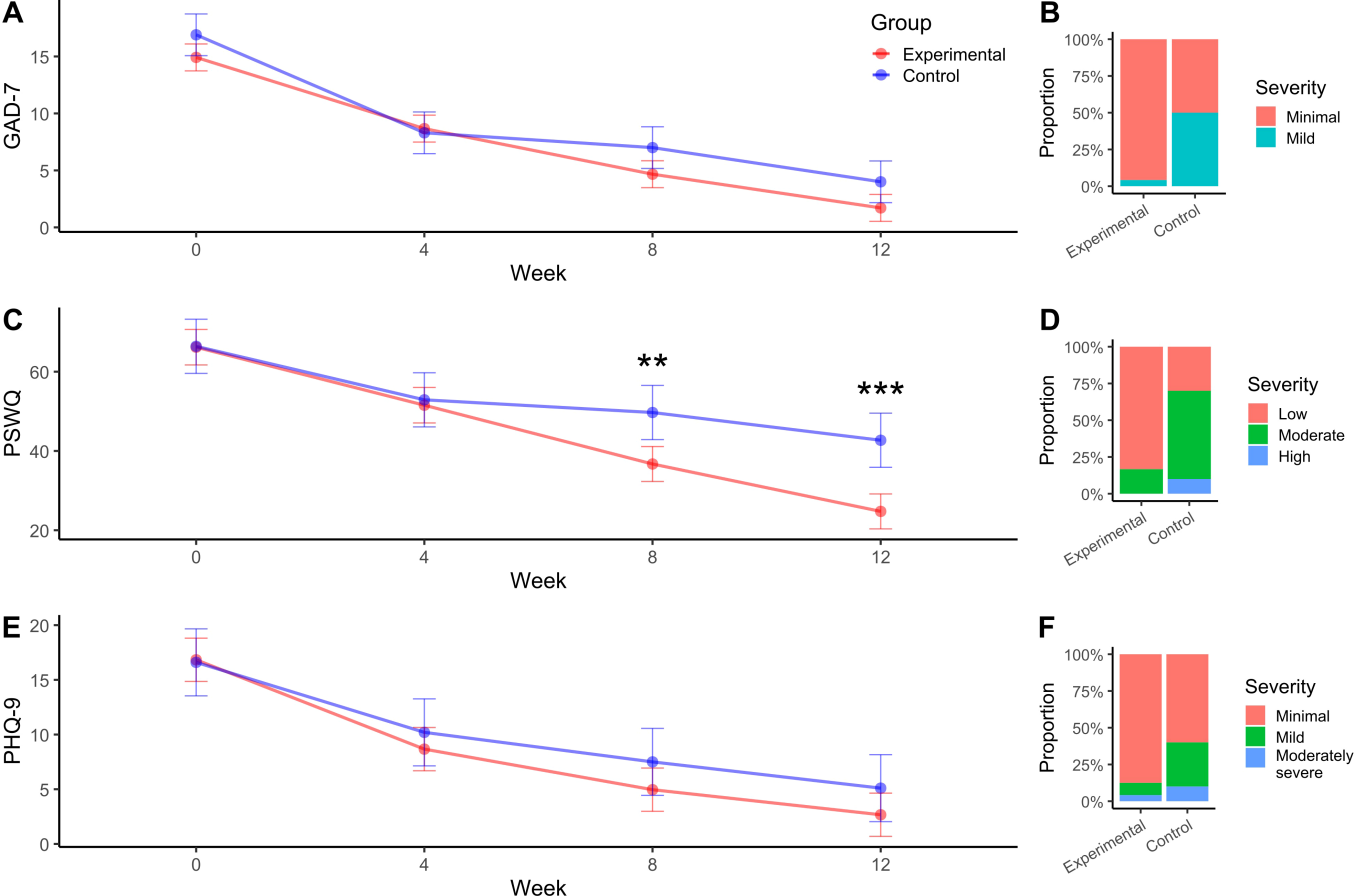
Longitudinal symptom trajectories and week-12 severity distributions for anxiety, pathological worry, and depression. **(A, C, E)** Temporal evolution of symptom severity on the GAD-7 **(A)**, PSWQ **(C)**, and PHQ-9 **(E)** scales over the 12-week intervention. Points represent estimated marginal means derived from linear mixed-effects models, and error bars indicate 95% confidence intervals (not standard deviation or standard error) at each assessment time point (weeks 0, 4, 8, and 12). Trajectories compare the experimental (Aurora + pharmacotherapy) and control (pharmacotherapy alone) groups. Statistical analyses included fixed effects for group, time, and group × time interaction, with subject-level random intercepts and false discovery rate (FDR) correction applied to pairwise comparisons. Across outcomes, both groups demonstrated significant improvement over time (week effects), with no statistically significant between-group differences in GAD-7 and PHQ-9 after correction, although numerical trends favored the experimental group at later time points. In contrast, PSWQ trajectories showed significant group and group × time interaction effects, with divergence emerging at week 8 and increasing at week 12. **Asterisks denote statistically significant between-group differences after FDR correction (**p < 0.01; ***p < 0.001). **(B, D, F)** Distribution of clinical severity categories at week 12 for anxiety **(B)**, pathological worry **(D)**, and depressive symptoms **(F)**. Bars depict the proportion of participants classified into severity categories for each group. Severity classification was based on established cut-offs for each scale, and analyses were performed using ordinal logistic regression and Fisher’s exact test. For GAD-7 and PSWQ, the experimental group demonstrated substantially higher odds of assignment to lower-severity categories (e.g., PSWQ: OR = 12.5; p = 0.004), with statistically significant differences in distribution confirmed by Fisher’s exact test (p = 0.005). In contrast, PHQ-9 severity distributions showed no significant differences between groups, consistent with the longitudinal analyses. Together, these panels provide complementary continuous (mean trajectories) and categorical (severity transitions) perspectives, highlighting that clinically meaningful improvements—particularly in pathological worry—may be more sensitively captured through severity-level shifts rather than mean score changes alone. All findings should be interpreted cautiously, given the pilot, non-randomized design and limited sample size.

Pairwise contrasts did not reveal significant between-group differences at any assessment after FDR correction, though numerical advantages for the experimental group emerged at weeks 8 and 12 (−2.3 points; p = 0.079). The magnitude of change observed in both groups is consistent with clinically meaningful improvement, although no clear incremental effect attributable to the intervention can be established within this pilot design. Overall, both groups experienced substantial reductions in anxiety, consistent with reports that digitally delivered CBT elements can yield clinically meaningful improvements even in brief or low-intensity applications (8, 17) (**Figure 1A**).

### 3.3 Anxiety severity at week 12

Categorical analyses revealed more pronounced group differences. Ordinal logistic regression indicated that participants in the experimental group had substantially greater odds of being classified into lower-severity categories at week 12 (β=3.14; p=0.009; OR = 23). Fisher’s exact test corroborated this distributional shift (p=0.005).

These results suggest a clinically meaningful shift in severity categories; however, given the non-randomized design and potential baseline imbalances, these findings should be interpreted as exploratory rather than confirmatory (**Figure 1B**).

### 3.4 Pathological worry trajectories (PSWQ)

Worry symptoms followed a distinct trajectory. Mixed-effects modelling demonstrated significant effects of Week (F_3,93.4_=79.18; p<0.001), Group (F_1,31.12_=6.96; p=0.013), and their interaction (F_3,93.4_=7.86; p<0.001). Between-group divergence emerged by week 8, with the experimental group showing substantially lower PSWQ scores (−13 points; p=0.004), widening further by week 12 (−18 points; p<0.001).

The magnitude of the observed reduction (13–18 points) is consistent with clinically meaningful improvement in pathological worry, suggesting a potential added benefit of the intervention in this cognitive domain. This pattern is consistent with mechanistic evidence suggesting that digital CBT modules targeting cognitive restructuring and worry-specific processes exert distinct and time-dependent effects on perseverative negative thinking (9, 11) (**Figure 1C**).

### 3.5 Worry severity at week 12

Severity analyses reinforced these findings. Ordinal regression showed significantly greater odds of lower severity classification for the experimental group (β=2.53; p=0.004; OR = 12.5), and Fisher’s exact test supported a substantially more favorable severity distribution (p=0.005).

These findings indicate a clinically relevant shift in severity categories, which may be particularly meaningful for clinical decision-making and risk stratification, although causal inference remains limited (**Figure 1D**).

### 3.6 Depressive symptoms (PHQ-9)

Depressive symptoms decreased significantly over time across both conditions (Week effect: F_3,96_ =57.07; p<0.001), yet neither the Group effect (p=0.242) nor the interaction (p=0.523) reached significance. Estimated marginal means confirmed no between-group differences at any time point.

These findings suggest that while depressive symptoms improved overall, the intervention did not demonstrate an additional measurable effect beyond pharmacotherapy within the 12-week timeframe (**Figure 1E**).

### 3.7 Depressive severity at week 12

Severity classification at week 12 showed a non-significant trend favoring the experimental group (β=1.48; p=0.092; OR = 4.4), but neither ordinal regression nor Fisher’s test (p=0.144) reached significance.

These results should be interpreted cautiously, as the study may be underpowered to detect clinically meaningful differences in depressive severity (**Figure 1F**).

### 3.8 Positive emotions across modules

Positive affect increased steadily across the nine intervention modules (Module effect: p<0.001). Neither the Item effect nor the Module × Item interaction was significant, and a reduced main-effects model provided an equally good fit (χ²=7.56, p=0.109). In the parsimonious model, Module effect remained highly significant (p<0.001), and Item also showed a significant main effect (p<0.001), indicating different average levels across items but no differences in their pattern of change.

**Figure 2:**
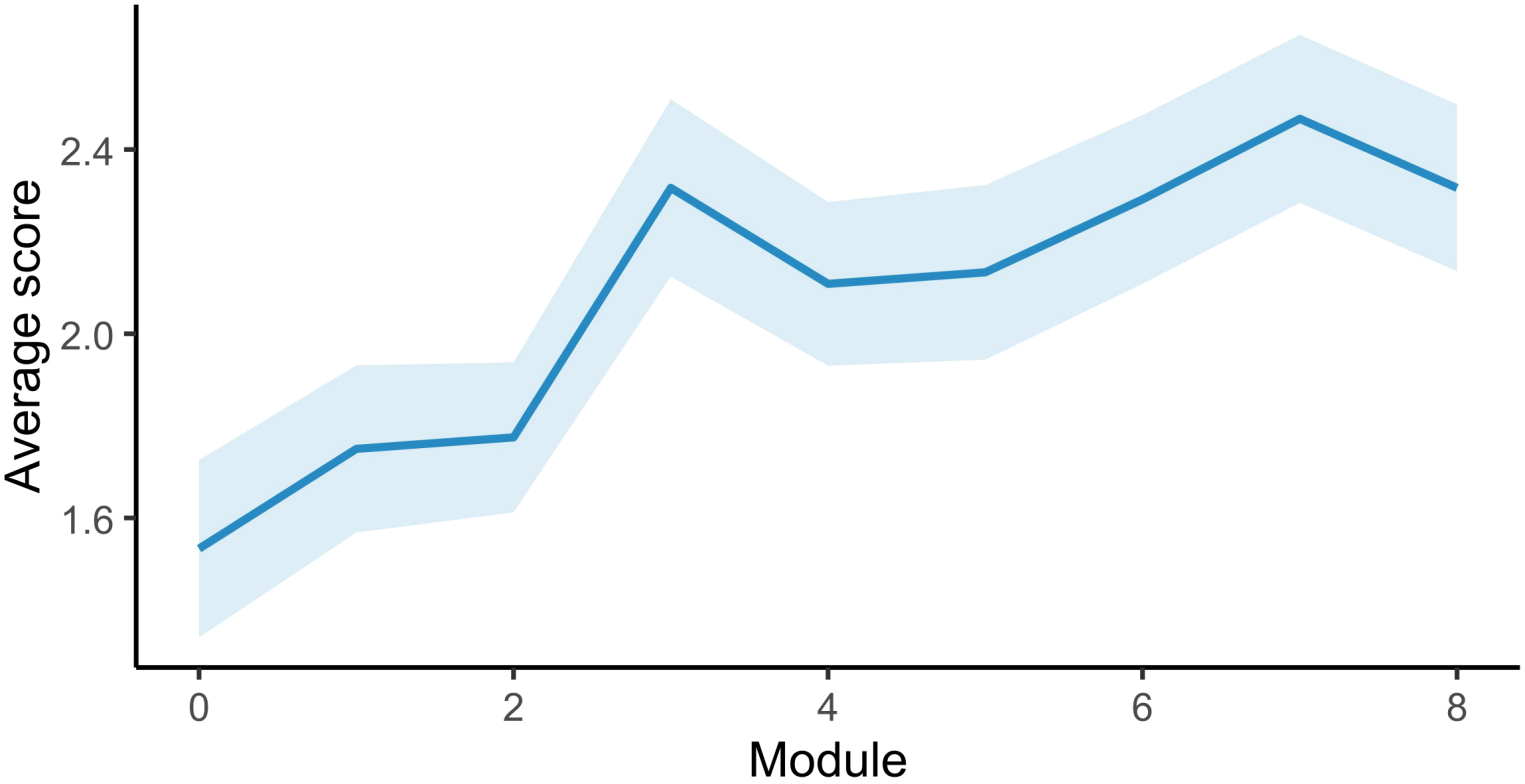
Trajectory of positive emotional states across intervention modules. Average scores for the five positive-emotion items are shown across modules 0–8. The solid line represents model-estimated mean values derived from linear mixed-effects models, and the shaded band indicates 95% confidence intervals. Statistical modelling included fixed effects for module and item, with subject-level random intercepts; a reduced model was selected based on likelihood ratio testing when interaction terms were non-significant. Results demonstrated a significant main effect of module (p < 0.001), indicating a progressive increase in positive emotional states across the intervention, with no significant module × item interaction, suggesting consistent changes across items. The upward trajectory reflects a robust, domain-general increase in positive affect, which aligns with contemporary mechanistic frameworks in digital CBT, in which enhancing positive emotional processing is linked to improved resilience and modulation of stress-related cognitive patterns. These findings should be interpreted as exploratory process-level outcomes, and while they support engagement with therapeutic content, their direct relationship with clinical improvement requires confirmation in larger, adequately powered studies.

These findings indicate a consistent and progressive increase in positive affect across modules, supporting engagement with the intervention’s core therapeutic components (**Figure 2**).

### 3.9 Associations with clinical improvement

Changes in positive emotion were not significantly correlated with changes in anxiety (r=0.28; p=0.193) or depressive symptoms (r=0; p=0.987). The association with change in pathological worry approached significance (r = -0.38; p = 0.071), suggesting that increases in positive emotion may preferentially influence cognitive domains characterized by repetitive negative thinking (22).

These exploratory associations should be interpreted cautiously due to limited statistical power and the study’s pilot nature.

### 3.10 Module-level anxiety- and depression-related cognitions

Module-embedded questions showed significant reductions from module 0 to module 8 for both anxiety-related (F_1,211_=41.51; p<0.001) and depression-related (F_1,215_=46.87; p<0.001) cognitions. Improvements were consistent across items and participants, indicating broad cognitive shifts aligned with CBT targets.

These results support the potential of the intervention to modulate cognitive processes targeted by CBT, although their relationship to clinical outcomes requires further validation.

### 3.11 Concurrent validity of module-based measures

At baseline, module-based anxiety and depression items correlated moderately with GAD-7 (r=0.48; p=0.018) and PHQ-9 (r=0.47; p=0.020), respectively, supporting concurrent validity. Post-intervention, depression-related items remained correlated with PHQ-9 (r=0.43; p=0.036), whereas anxiety-related items showed attenuation of association with GAD-7.

Change–change correlations were non-significant, a pattern commonly observed due to the lower reliability of difference scores and the multifactorial nature of clinical improvement (23).

These findings suggest that in-app measures may provide complementary—but not interchangeable—information relative to traditional clinical scales, particularly as symptom severity decreases.

## 4 Discussion

This controlled pilot study provides preliminary, hypothesis-generating evidence that a culturally tailored, self-guided digital therapeutic—Aurora—can be safely integrated with pharmacotherapy and may be associated with improvements in anxiety-related outcomes, particularly pathological worry, in a Mexican clinical population. Across multiple analytic frameworks, the intervention demonstrated a favorable safety profile, substantial reductions in anxiety and pathological worry, and improvements in severity level. However, given the non-randomized design and limited sample size, these findings should be interpreted cautiously and not as definitive evidence of efficacy. These results nonetheless point toward the feasibility of integrating digital CBT into psychiatric care pathways in resource-constrained systems and contribute to a broader re-imagining of mental health delivery in Latin America. Importantly, the favorable safety profile—including the absence of serious adverse events and no difference in adverse-event burden between groups—should be considered preliminary, as the sample size (n=34) limits the ability to detect rare or delayed adverse effects.

The improvement patterns observed here reflect emerging mechanistic insights from digital CBT research. In particular, the early and persistent reduction in pathological worry echoes findings from trials demonstrating that digitally delivered cognitive restructuring can specifically target perseverative negative thinking—a transdiagnostic process central to generalized anxiety disorder (9, 11). Moreover, the steady increase in positive affect across modules aligns with neurobehavioral models in which enhanced positive emotion serves as a resilience mechanism that attenuates stress-responsive circuits and improves emotion regulation (17, 19). These findings suggest that Aurora extends beyond symptomatic relief; it may facilitate the recalibration of core cognitive–affective processes that perpetuate anxiety and depressive symptomatology. Notably, the magnitude of change observed in pathological worry (13–18 points) and the substantial increase in the odds of lower severity classification (OR = 12.5) indicate clinically meaningful shifts, supporting the potential relevance of digital CBT for targeting core cognitive domains not fully addressed by pharmacotherapy alone. The significant reductions observed in the brief cognitive items embedded within Aurora’s modules further support this interpretation, indicating early shifts in appraisal patterns that correspond to core CBT targets.

The findings also underscore the broader promise of digital medicine in middle-income countries, where traditional mental health systems struggle to match demand. Mexico illustrates this challenge acutely: despite a high prevalence of common mental disorders, the country faces persistent workforce shortages, financial barriers, and fragmented service delivery (3, 4). Our study builds on the first Aurora feasibility evaluation, which demonstrated high usability and clinically meaningful pre–post symptom reductions among pharmacologically treated adults (16).

Together, these findings position Aurora as a prototype for locally engineered, clinically evaluated digital therapeutics designed for Latin American contexts. In contrast to generic commercial apps, Aurora was co-created with clinicians and patients, ensuring therapeutic fidelity and cultural alignment from inception—a model increasingly recognized as essential for global digital health equity (13, 24). This co-design strategy may also underlie the favorable engagement patterns observed across modules, as cultural and linguistic adaptation has repeatedly been shown to enhance usability and adherence in digital mental health interventions (25). Importantly, integrating adherence metrics—such as module completion and in-app engagement—into the analytical framework provides additional support for the feasibility of sustained user engagement in real-world clinical settings. The results of this study suggest that locally designed digital interventions can be feasibly deployed in clinical environments, supporting stepped-care or hybrid-care approaches that are increasingly viewed as essential for expanding access in resource-constrained systems (26, 27).

Beyond local relevance, this study contributes to an international debate on how digital therapeutics can reshape mental health care ecosystems. Evidence from randomized trials in Brazil, Peru, and South Asia shows that smartphone-based CBT can produce clinically significant improvements even when deployed in strained health systems (11, 12). Our results extend this line of evidence by demonstrating that digital therapeutics may function not only as substitutes for psychotherapy but also as complementary interventions, although the present study design does not allow causal inference regarding additive effects. The combined approach—digital plus medication—has been proposed as a next-generation model for scalable psychiatric care (17, 20). The observed differential effect on pathological worry, in contrast to the absence of additional benefit for depressive symptoms, suggests domain-specific responsiveness that warrants further investigation in adequately powered randomized trials.

Several methodological limitations must be explicitly acknowledged. First, the non-randomized, convenience-based allocation introduces potential selection and allocation bias, limiting internal validity and precluding causal inference. Second, baseline imbalances—including differences in sex distribution—may have influenced outcomes despite the use of longitudinal mixed-effects models, and residual confounding cannot be excluded. Third, the absence of an active control condition limits the ability to disentangle the specific therapeutic effects of the digital intervention from non-specific factors such as patient expectations, engagement, or attention-related effects. Fourth, the small sample size (n=34) restricts statistical power and increases the risk of both type I and type II errors, limiting generalizability. Fifth, outcomes relied primarily on self-reported measures (GAD-7, PSWQ, PHQ-9), which may be influenced by reporting bias and repeated-measurement effects, while in-app metrics—although promising—require further validation as digital biomarkers. Finally, the 12-week follow-up does not allow assessment of long-term durability of response or delayed adverse events.

In addition, the study population was predominantly urban and relatively well-educated, raising important considerations for scalability and equity. Future implementation efforts must address barriers to digital literacy, mobile technology access, connectivity, and linguistic diversity to ensure that digital therapeutics do not inadvertently widen existing disparities in mental health care.

Despite these limitations, the clinical implications of this work are noteworthy. The convergence of symptom improvement, severity-level shifts, and engagement metrics suggests that digital CBT may offer clinically meaningful benefits, particularly in domains such as pathological worry that are central to generalized anxiety disorder. Importantly, the use of severity-level transitions and ordinal modelling provides a more clinically interpretable framework, aligning outcomes with real-world decision-making processes rather than relying solely on mean score changes.

The modular architecture of Aurora, combined with its usability and engagement patterns, provides a potential framework for delivering structured CBT at scale while preserving therapeutic fidelity. These findings support a gradual transition toward hybrid models of care, in which digital interventions complement pharmacotherapy and extend the reach of evidence-based treatments within routine clinical practice.

If validated in larger, well-controlled randomized trials, Aurora and similar interventions could become integral components of modern psychiatric care in Mexico and other LMIC/UMIC contexts. Ongoing randomized studies will be essential to confirm efficacy, establish causal relationships, and define the role of digital therapeutics within stepped-care and precision psychiatry frameworks. For health systems facing persistent treatment gaps and increasing demand, digital therapeutics represent a promising—but still evolving—strategy toward a more equitable, scalable, and responsive model of mental health care.

## Data Availability

The original contributions presented in the study are included in the article/**Supplementary Material**. Further inquiries can be directed to the corresponding authors.
